# Phase III Randomized Pair Comparison of a Barrier Film vs. Standard Skin Care in Preventing Radiation Dermatitis in Post-lumpectomy Patients with Breast Cancer Receiving Adjuvant Radiation Therapy

**DOI:** 10.7759/cureus.4807

**Published:** 2019-06-03

**Authors:** Andrew CL Lam, Edward Yu, Danielle Vanwynsberghe, Melissa O'Neil, David D'Souza, Jeffrey Cao, Michael Lock

**Affiliations:** 1 Radiation Oncology, University of Toronto, Toronto, CAN; 2 Oncology, Schulich School of Medicine and Dentistry, Western University, London, CAN; 3 Radiation Oncology, London Regional Cancer Program, Western University, London, CAN; 4 Radiation Oncology, London Regional Cancer Program, Western University, London, CAN; 5 Radiation Oncology, Schulich School of Medicine and Dentistry, Western University, London, CAN

**Keywords:** breast cancer, dermatitis, skin, barrier, radiation

## Abstract

Introduction

Patients undergoing adjuvant radiotherapy to the breast often experience radiation dermatitis ranging from mild erythema to moist desquamation. In post-lumpectomy patients, the axilla and inframammary fold are at an increased risk for friction dermatitis. Dermatitis can impact patients’ quality-of-life and may require treatment break/cessation. Our objectives are to assess the efficacy of 3M Cavilon Barrier Film (BF) in preventing and/or delaying the onset of grade-two dermatitis and reducing patient-reported sensation scores.

Methods

A total of 55 patients were randomized to receive BF on the medial or lateral breast. BF was applied twice weekly during treatment. Skin toxicity was evaluated weekly by a blinded clinical investigator using the Skin Toxicity Assessment Tool (STAT) and the modified Radiation Therapy Oncology Group Visual Assessment Score (RTOG VAS). On day one, baseline photographs were taken; seven-to-ten days post-treatment, patients returned for photographs, the STAT/RTOG VAS, and patient-opinion questions in the form of the global questionnaire.

Results

The paired analysis found BF did not significantly reduce dermatitis either during or post-treatment. However, the unpaired analysis found significantly reduced RTOG VAS on the lateral compartment during treatment (BF:0.91 vs. Control:1.21, *p* = 0.0408). This difference resolved post-treatment. Additionally, BF was able to reduce pruritus (*p* = 0.047) on the medial components and burning sensations on the lateral components (*p *= 0.035). There was no significant difference between the time-to-onset or proportion of patients who developed grade-two dermatitis.

Conclusion

In an unpaired analysis, BF significantly reduced dermatitis on the lateral compartment during treatment. Additionally, BF significantly reduced pruritus and burning sensations. A larger study using a more reliable scoring method is required to clarify the effect of BF on radiation-associated skin toxicity.

## Introduction

Patients undergoing adjuvant radiotherapy to the whole breast frequently experience radiation dermatitis in the treatment area. Radiation affects both cancer cells and healthy cells resulting in radiation-induced skin reactions. These skin reactions can vary in severity ranging from erythema to moist desquamation. Skin reactions can also be accompanied by varying degrees of tenderness, pruritus, pulling, and/or burning sensations. After a lumpectomy, the intact breast and surrounding areas (especially the axilla and inframammary fold) are at high-risk for friction trauma. The movement of the breast, arm, and skinfolds can cause friction trauma which can be further exacerbated by moisture trapping that can occur in these areas.

There are many prescription and over-the-counter creams/ointments available for the management of moist desquamation. However, a 2014 systematic review of 47 studies concluded that, although there have been trials that demonstrated the efficacy of certain products, there is limited high-quality evidence that a single product is able to reduce radiation-induced skin reactions [1]. The objective of this investigation is to assess the efficacy of 3M Cavilon Barrier Film (BF) as a prophylactic agent in preventing grade two radiation dermatitis in post-lumpectomy breast cancer patients. BF is an alcohol-free film formulated from two polymers. Acrylate terpolymer provides durability to the film; methylphenyldisiloxane acts as a plasticizing agent to provide flexibility and prevent cracking. When applied to the skin surface, the film creates a barrier which protects against friction trauma, allows time for repopulation of epidermal stem cells (thus avoiding moist desquamation), and maintains skin hydration while still permitting the skin to breathe [2]. By protecting the irradiated skin from friction trauma, we hypothesize that radiation-induced skin toxicity can be minimized and the onset delayed.

A 2004 paired randomized control trial by Graham et al. compared the prophylactic use of BF vs. 10% sorbolene cream (glycerin, paraffin soft, and mineral oils) in post-mastectomy patients receiving adjuvant radiation. In this trial, patients were randomized to either medial or lateral BF application with intra-individual paired comparison, scored using the modified Radiation Therapy Oncology Group Visual Assessment Score. The study reported a non-significant reduction in grade two dermatitis with BF (33%) compared to sorbolene (48%) as well as a significant reduction in pruritus (*p *= 0.019) [3].

However, the majority of breast cancer patients undergo lumpectomies instead of mastectomies and no study to date has yet to specifically investigate the ability of BF to reduce toxicity in post-lumpectomy patients. This trial will evaluate the efficacy of prophylactic BF application in reducing skin toxicity in post-lumpectomy patients, a patient population that has a lower incidence of moist desquamation when compared to post-mastectomy patients [4].

## Materials and methods

This trial was approved by the University of Western Ontario Research Ethics Board (103026) and registered in the National Institute of Health trial registry ClinicalTrials.gov (NCT01762020). All patients were recruited from and treated at the London Regional Cancer Program, a tertiary cancer center. Written informed consent was obtained from patients prior to study entry.

Patients and radiation treatment

Inclusion criteria limited accrual to women aged 18-90 who had undergone a lumpectomy and had been prescribed a standard dose (42.5 Gy in 16 fractions or 50 Gy in 25 fractions) of adjuvant tangential radiotherapy, without the need for a boost or bolus. Radiation to the breast was administered using 6 MV, 10 MV, 18 MV, or a weighted combination of both 6 MV and 18 MV. Beams were applied using a forward planned segmented field-in-field tangential technique. The maximum acceptable dose was 110%.

Application of BF and Glaxal Base cream

**Figure 1:**
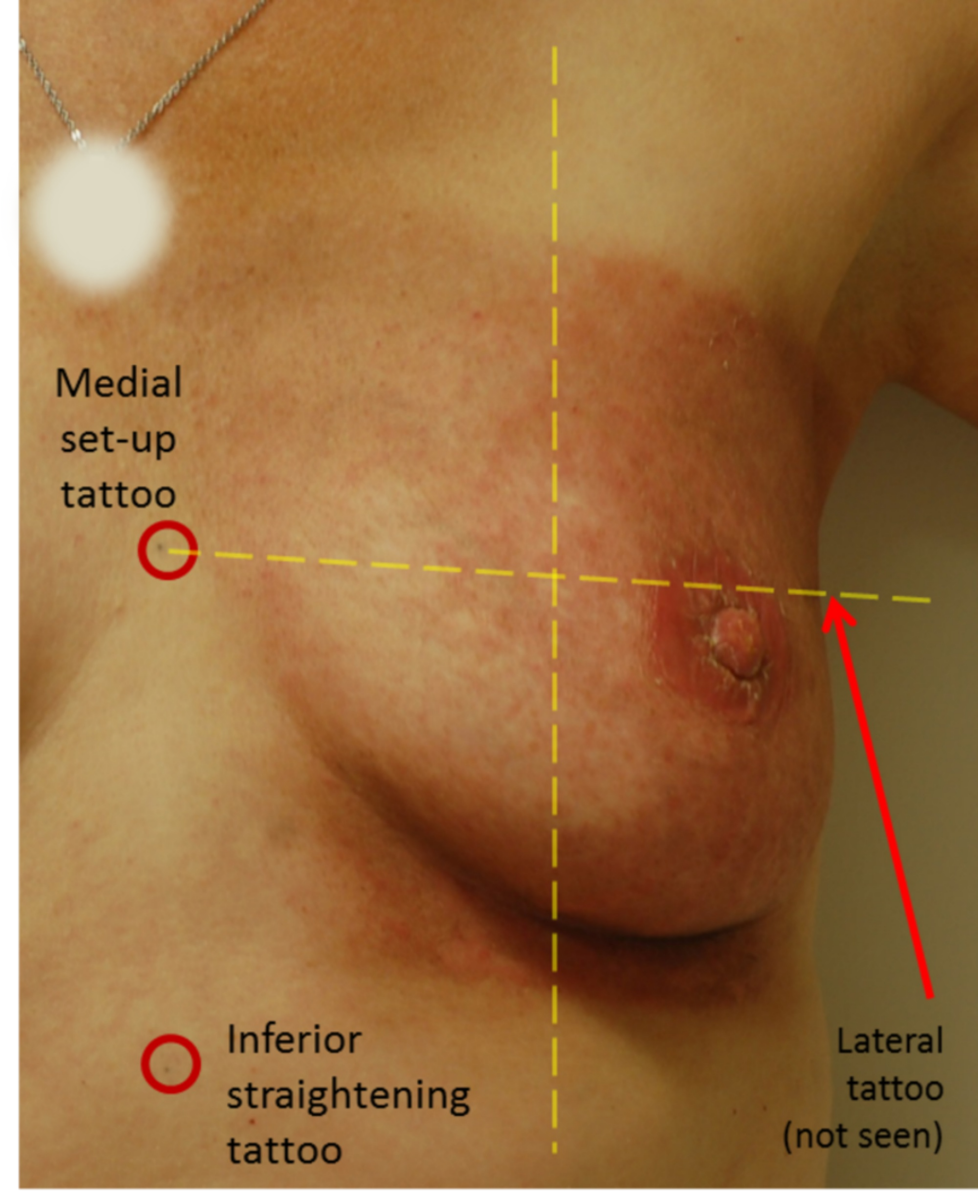
Breast Division Breast division into superior and inferior halves and medial and lateral halves created four quadrants. The patient was photographed when upright but delineation occurred when supine. Barrier film was applied to the medial half and standard-of-care on the lateral half for this patient.

The post-lumpectomy breast was divided into lateral and medial halves based on a volume midline measurement determined at computed tomographic simulation. Patients were then randomized in a one-to-one ratio to either receive BF in the lateral half of the breast or the medial half of the breast. The half that did not receive BF received the standard-of-care (SOC) and served as an intra-individual comparison. In the SOC half of the breast, patients were educated on our center’s standard-of-care skin care practices, which includes the use of Glaxal Base Cream (GBC). GBC is a non-medicated cream formulaically similar to aqueous cream, which has been shown to have no effect on the development of radiation dermatitis [5]. Randomization was done using a random allocation list generated using SAS (SAS Institute, Cary, NC) with two stratification blocks (chemotherapy vs. no chemotherapy and breast separation). The patient and the radiation therapist administering the film were not blinded to treatment allocation, but all clinical investigators tasked with scoring skin reactions and/or administering the questionnaires were blinded. Solely for assessment purposes, the breast was further subdivided into superior and inferior halves which, when combined with lateral and medial halves, created four quadrants (Figure 1). 

BF application started on day one of radiotherapy and continued until treatment completion. Application occurred twice per week by radiation therapists in the treatment unit to ensure compliance and correct application technique per product instructions. BF was not applied in the period between the patient’s last radiation treatment and her post-treatment follow-up appointment. The line delineating medial and lateral components was dotted on the patient’s skin to aid in application accuracy and to guide the patient’s application of GBC should she choose to use it on the SOC half of the breast. When clinically indicated, BF and/or the SOC application was stopped on areas that developed brisk erythema requiring a topical anti-inflammatory cream (e.g., 1% hydrocortisone) or areas that developed moist desquamation requiring a topical antibacterial cream (e.g., silver sulfadiazine). The presence of grade two dermatitis would always precede treatment stoppage and dermatitis would be scored and recorded prior to the application of alternative treatment. As a result, the application of antibacterial and/or anti-inflammatory cream did not interfere with our primary outcome. 

Scoring of skin toxicity

Each quadrant was evaluated for skin toxicity on a weekly basis by a blinded clinical investigator using the Skin Toxicity Assessment Tool (STAT). The STAT is a validated method of capturing different aspects of skin toxicity and consists of three sections: patient and treatment characteristics, objective scoring of skin reactions, and patient-reported sensation scores [6]. In this study, the objective scoring of skin reactions section was supplemented with the modified Radiation Therapy Oncology Group Visual Assessment Score (RTOG VAS) (Table 1) [7-8]. Patient-reported sensation scores were divided into four categories: burning sensation, pruritus, tenderness, and pulling-sensation. Patients would then rate these sensations on a zero to five visual analog scale.

**Table 1:** Modified Radiation Therapy Oncology Group Visual Assessment Score The modified Radiation Therapy Oncology Group Visual Assessment Score is a clinician-assessed scoring criterion used to quantify the severity of radiation-induced dermatitis [7-8].

Radiation Therapy Oncology Group Visual Assessment Score	Skin Changes
0	No changes over baseline
1	Follicular, faint, or dull erythema
1.5	Dry desquamation
2	Tender or bright erythema
2.5	Patchy moist desquamation
3	Confluent, moist desquamation
4	Ulceration, hemorrhage, necrosis

On day one of treatment, baseline photographs were taken. Of the weekly scores, the highest was taken as the score during treatment. Seven-to-ten days post-treatment, patients returned for a final skin assessment using the STAT and RTOG VAS, the second set of photographs, and completion of the global questionnaire (GQ). The GQ consist of two opened-ended questions which examined the patient’s personal opinion of BF (“Would you use BF again? Why?” and “Did BF make a meaningful difference in your skin care?”). Each photograph was assigned an RTOG VAS by each of the three blinded raters.

Study outcomes

Our primary objective was to evaluate the effectiveness of BF in preventing grade two radiation dermatitis induced by adjuvant radiotherapy in post-lumpectomy patients. Secondary objectives included an evaluation of BF in reducing patient-reported sensations, impressions of the overall effectiveness of BF from the patients’ perspective, and improvements in the time-to-onset of grade two radiation dermatitis. Time-to-onset of radiation dermatitis was calculated in days from the date at which the first dose of radiation was delivered until the date of assessment when the grade two reaction was first noted. Although in some cases, the date of the first weekly assessment did not coincide with the first dose of radiation treatment, the first assessment would have at least occurred within the first five fractions. Due to cellular mechanisms underlying a radiation-induced skin reaction and the cumulative dose required to induce skin toxicity, it is reasonable to assume that an RTOG VAS of zero would precede the first scoring opportunity.

Statistical analysis

Based on previous trials, we estimated the SOC risk of grade two toxicity to be approximately 60% at any point during the assessment period [3,8]. Studies on the same product demonstrate an expected reduction of grade two toxicity of approximately 30% [3]. Assuming a Type I error of 5% and a power of 80% to reject the null hypothesis, the sample size required was 45. With an expected 15% excess added due to loss to follow-up or non-evaluable data, we required sample size of 53 patients.

To compare continuous data, the Wilcoxon rank-sum test was used when unpaired and the Wilcoxon signed-rank test was used when paired. McNemar’s test was used to compare categorical data when paired and a Chi-square test was used when unpaired. A Prentice-Wilcoxon test was used to analyze paired time-to-onset [9]. The RTOG VAS for the photographs were analyzed using a three-way ANOVA with a Tukey post hoc test; treatment, compartment, and rater were factors. All data analysis was performed using R3.2.3 and all tests were two-sided with a significance level of 5%.

## Results

**Figure 2:**
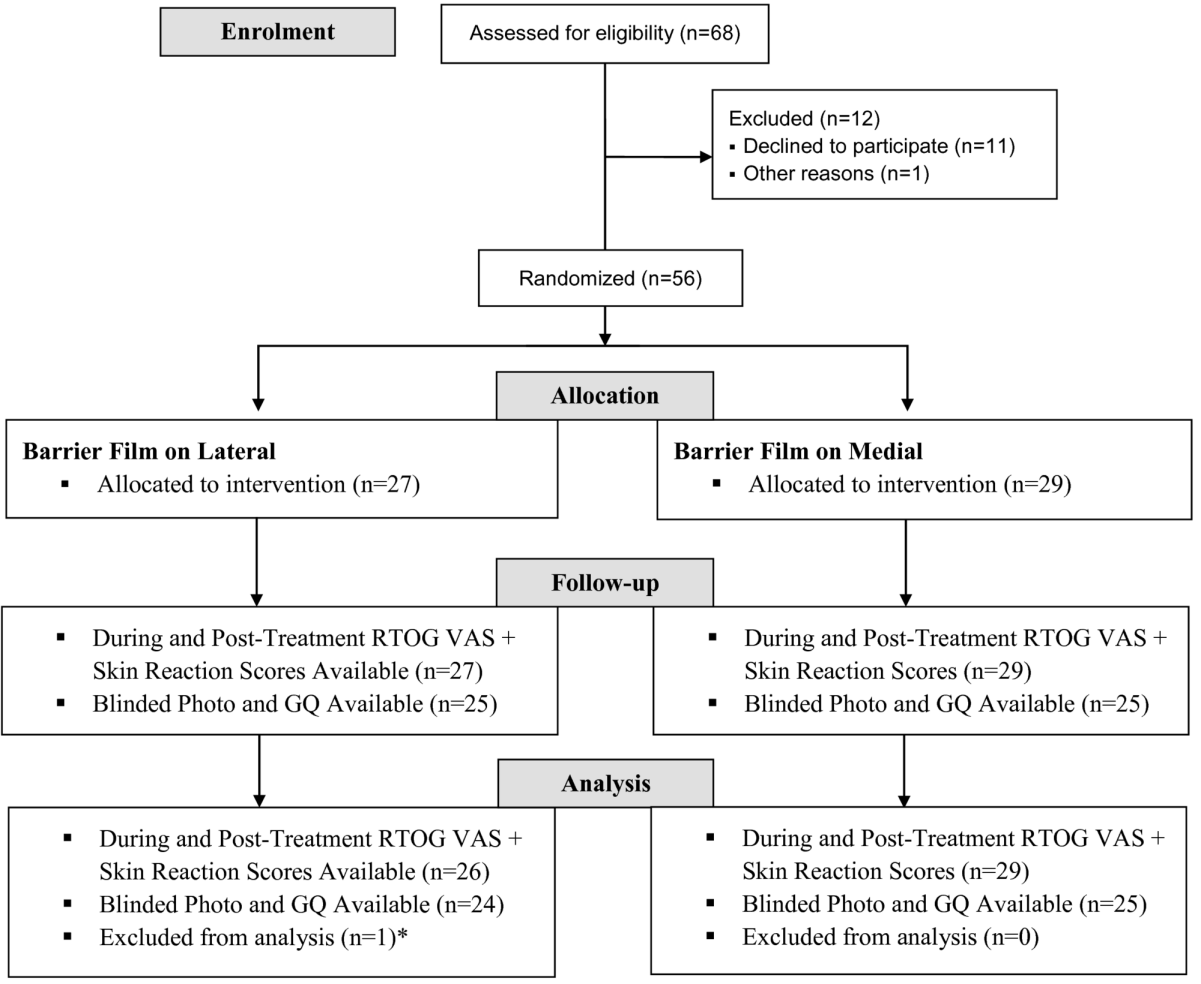
Patient Flow Through Phase III Trial *Patient applied standard-of-care cream over top of the barrier film from the beginning of the trial. This affects the integrity of barrier film and as a result, she was excluded from the analysis. RTOG VAS = modified Radiation Therapy Oncology Group Visual Assessment Score, GQ = Global Questionnaire.

Patient recruitment occurred between 2013 and 2015, and it concluded when the prespecified sample size was reached. RTOG VAS and STAT scores were available for 55 patients. Post-treatment photographs and GQ responses were available for 49 patients (Figure 2). Patient characteristics, diagnosis, and treatment are outlined in Table 2.

**Table 2:** Patient Demographics, Treatment Characteristics, and Breast Cancer Stage All patients had margins ≥2 mm and no nodes were involved. All patients received sentinel node or axillary dissection. BF = Barrier Film.

	BF on Lateral (n = 26)	BF on Medial (n = 29)	Total (n = 55)
Patient Characteristics				
Mean age (years)	62.7	61.8	62.1
Age range (years)	47-86	45-78	45-86
BMI mean	29.6	31.8	30.7
Treatment Characteristics				
Dose mean (Gy)	45.2	45.4	45.4
Fraction mean	19.4	18.9	19.4
Photon Energy	6, 17, 2, 1,	8, 17, 2, 2	14, 34, 4, 3
6, 6/18, 10, 18 (%)	(23.2, 65.3, 7.7, 3.8)	(27.6, 58.6, 6.9, 6.9)	(25.4, 61.8, 7.3, 5.5)
Hormone n(%)	13 (50.0)	13 (44.8)	26 (47.3)
Chemotherapy n(%)	6 (23.1)	3 (10.3)	9 (16.4)
Breast Cancer Stage				
Tis	4	7	11
T1	15	14	29
T1mi	0	1	1
T1a	2	0	2
T1b	1	2	3
T1c	2	3	5
T2	1	2	3
T3	1	0	1

**Figure 3:**
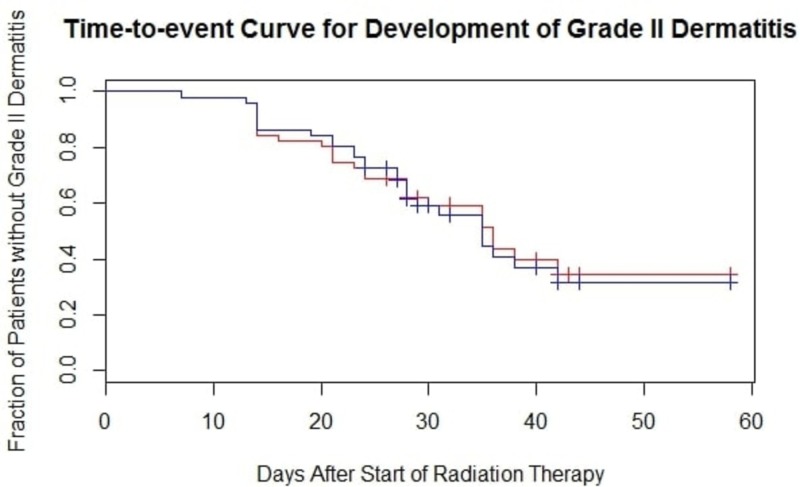
Time-to-event Curve for the Development of Grade Two Dermatitis The red line represents barrier film regions and the blue line represents standard-of-care regions (*p = *0.89). Statistical significance was determined using a Prentice-Wilcoxon test.

Paired analysis of the RTOG VAS obtained during and post-treatment found no significant difference between BF and the SOC. However, in an exploratory analysis, the lateral halves treated with BF had a significantly lower RTOG VAS during treatment than lateral halves treated with the SOC (*p *= 0.041). This difference was resolved post-treatment. The use of BF in the medial halves led to non-significantly higher RTOG VAS during treatment but led to a lower RTOG VAS post-treatment. Table 3 summarizes unpaired RTOG VAS obtained during treatment and post-treatment by intervention and compartment; grade 2 is represented as 2-2.4 and grade 2.5 is represented as 2.5-2.9. There was no significant difference between the incidence of grade two dermatitis (*p *= 0.79 during treatment, *p *= 0.50 post-treatment) and in an exploratory analysis, there was no difference in grade two-point-five dermatitis (moist desquamation) (*p *= 1.00 during and post-treatment). There was no significant difference in the time-to-onset of grade two dermatitis between BF and SOC treated halves, shown in Figure 3 (*p *= 0.89).

**Table 3:** Summary of RTOG VAS During and Post-treatment (Unpaired) The analysis was performed using the Wilcoxon rank-sum test. BF = Barrier Film, RTOG = Radiation Therapy Oncology Group, SOC = Standard-of-Care.

Scores During Treatment												
		RTOG Visual Assessment Scores										
Compartment	Treatment	0-0.9	1-1.9	2-2.4	2.5-2.9	3	Mean Score	p-value	≥ Grade 2 n(%)	Total		
Lateral	BF	20	23	5	3	1	0.91	0.041	9(17.3)	52		
	SOC	7	36	13	2	0	1.21		16(27.6)	58		
Medial	BF	14	35	8	1	0	0.96	0.76	10(17.2)	58		
	SOC	20	27	3	1	1	0.88		5(9.6)	52		
Scores Post-treatment												
		RTOG Visual Assessment Scores										
Compartment	Treatment	0-0.9	1-1.9	2-2.4	2.5-2.9	3	Mean Score	p-value	≥ Grade 2 n(%)	Total		
Lateral	BF	5	24	10	9	4	1.59	0.63	23(44.2)	52		
	SOC	9	23	22	11	3	1.50		26(44.8)	58		
Medial	BF	8	34	12	3	1	1.24	0.29	16(27.6)	58		
	SOC	6	27	15	3	1	1.38		19(36.5)	52		

Paired analysis within the four sensation categories yielded no significance both during and post-treatment. However, an exploratory unpaired analysis of post-treatment scores for burning sensations on the lateral compartments (*p *= 0.047) and pruritus on the medial compartments (*p *= 0.035) were significantly lower when BF was applied, as summarized in Table 4. With the exception of burning and tenderness on the medial compartments, compartments treated with BF had lower sensation scores; although, many of these scores never reached the level of significance.

**Table 4:** Patient Sensation Scores One-week Post-treatment (Unpaired) Patients rated sensations on a six-point Likert scale. Analysis was performed using Wilcoxon’s rank-sum test. BF = Barrier Film, SOC = Standard of Care.

Compartment	BF Mean vs. SOC Mean (p-value)			
	Burning	Itching	Pulling	Tenderness
Lateral	0.92 vs 1.83 (0.047)	0.85 vs 1.02 (0.39)	0.79 vs 1.09 (0.52)	1.77 vs 2.03 (0.55)
Medial	0.62 vs 0.52 (0.87)	1.14 vs 2.06 (0.035)	0.12 vs 0.19 (0.93)	1.57 vs 1.17 (0.62)

There was no significant difference in RTOG VAS obtained from photographs between treatments. However, there was a significant difference between the three raters (*p *< 0.01 between all three raters) and a significantly higher RTOG VAS for lateral halves compared to medial halves (*p *< 0.01). Further analysis revealed that the intraclass correlation coefficient between the three raters was 0.45. At the end of the study, 77% of patients said they would use BF again while 58% felt it made a meaningful impact on their skincare routine. Of the patients who would not use BF again, frequently cited concerns include the long drying time, potential cost, and the inability to use other products in conjunction with BF. Patients who stated BF made a meaningful impact frequently stated that BF led to a reduction in erythema, less dry skin, and liked that BF did not require frequent application.

## Discussion

It is estimated that 95% of patients undergoing radiotherapy will experience changes to their skin; these changes may limit the dose a patient can receive [10]. Skin-related side-effects of radiotherapy can be painful and debilitating, yet there is no clear consensus for the best practices for skin care [1]. Most studies investigating breast cancer skin-management after radiotherapy have focused on products which are moisturizing, antimicrobial, or anti-inflammatory. Few have focused on preventing friction-related damage and even fewer have focused specifically on post-lumpectomy patients [11]. Friction removes the top layer of the epidermis at a rate that the radiation-damaged epidermal stem cells cannot keep up with. The axilla, lateral compartment of the breast and inframammary fold are subjected to higher moisture levels due to sweat and moisture retention. The increased moisture, compounded with higher friction forces, leads to decreased skina integrity [12]. BF, being water-impermeable and friction reducing, is thought to be able to maintain the barrier property of the skin, facilitate healing, and stabilize wound margins [2,12].

In this study, we compared BF with the current SOC in post-lumpectomy patients receiving adjuvant radiotherapy. Paired-analysis revealed no significant difference in BF’s ability to reduce grade two dermatitis, patient-reported sensation scores, or time-to-onset of grade two dermatitis either during treatment or post-treatment. Past studies have yielded mixed results. A study investigating Mepitel Film, a silicone dressing with similar friction reducing properties as BF, found it effective in reducing skin reaction (*p *< 0.0001) and rates of moist desquamation (*p *< 0.001) [13]. Their study reported a 46% incidence of grade two-point-five dermatitis in the control arm compared to 8% in the Mepitel arm. In contrast, we found incidence to be the same in both control and BF arms at 16%. The large variation in the incidence of grade two-point-five dermatitis in the control arm of both studies suggests that there are large variations between cancer centers, likely due to patient and/or treatment factors [14]. Similarly, a 2004 study performed by Graham et al., found that BF was able to reduce dermatitis (*p *= 0.005) and pruritus (*p *= 0.011) [3]. However, when Graham et al. conducted a double-blinded version of the 2004 study with a larger sample size in 2013, no significant difference was found [15]. This may be attributed to the study using a different formulation, for the purposes of double-blinding, compared to the previous 2004 trial. This highlights the difficulty in double-blinding BF trials as the appearance and application of BF is drastically different than other products. Finally, a 2015 study performed by Shaw et al., comparing BF with corticosteroids and with no intervention, found no significant difference in BF’s ability to reduce time-to-onset of radiation dermatitis [16]. However, this study was limited by its small sample size.

Although the paired analysis did not reveal any significant differences, the unpaired analysis found that BF was able to significantly reduce skin toxicity during treatment in the lateral compartment. Additionally, BF significantly reduced burning-sensations and pruritus in the lateral and medial compartments, respectively. Compared to the medial compartment, the lateral compartment of the breast experiences increased friction from the underside of the arm and articles of clothing. For scoring purposes, the axilla was included in the lateral breast. It is possible that the application of BF reduced the friction the lateral compartment experienced, which may explain the protective effects seen during treatment compared to the SOC. The medial compartment, which is not subjected to the same friction forces, did not receive the same benefit. Similar to our study, both studies performed by Graham et al. also noted that there was a larger difference between compartments than between treatments [3,15].

The first limitation is the discontinuation of BF application between the patients’ last treatment session and their post-treatment appointment; the post-treatment appointment was when post-treatment scores were obtained. BF is clinically proven to last 72 hours only, after which the film degrades [2]. During this time, skin changes may occur and can occur up to three months post-treatment [17]. This may explain why the protective effects, seen during the treatment when BF was continuously applied, did not extend post-treatment. In past studies, BF and its comparison were continuously applied one-to-two weeks post-treatment [3,15,17]. In our study, the application of BF was performed exclusively by a radiation therapist in order to ensure accurate application and proper technique. As a result, BF application between the patients’ last treatment and their post-treatment appointment was not feasible as patients were not scheduled to return during this time.

The second limitation of this study was the low inter-rater reliability seen with the scoring of skin reactions. Although the STAT has been validated in its ability to capture skin toxicity, it suffered from low inter-rater reliability in the objective scoring of skin reactions section in the validation study [6]. In an effort to improve reliability, reduce variation, while still accurately capturing skin toxicity, weekly scoring was only performed by two radiation therapists and the objective scoring section was supplemented with the RTOG VAS. The RTOG VAS is widely used and correlates well with biophysical changes to the skin [3,7,13,18-19]. When compared to the unmodified scale, the modified RTOG VAS scale grades bright erythema as two and moist desquamation as two-point-five, as opposed to grouping both into a score of two. Moist desquamation, where the integrity of the skin is compromised, is considered more severe than erythema [7]. However, compared to the three-tiered erythema scale used in the STAT, the RTOG VAS uses a seven-tiered scale. Although this allows for more aspects of skin toxicity to be captured, it may further exaggerate the discordance between raters. Future studies may consider scales such as the Radiation-Induced Skin Reaction Assessment Scale which allow clinicians to separately grade erythema, dry desquamation, moist desquamation, and necrosis based on presence and surface area [20]. Other alternatives include the National Cancer Institute Common Toxicity Criteria for Adverse Events and the Catterall skin scoring profile, a ten-point scale that ranges from no visible reaction to ulceration [21-22]. Additionally, spectroscopy and software programs have been used to reliably score each patient, though this method is more time and resource intensive [23-24].

Third, this study originally planned to just use an intraindividual paired comparison for each patient. However, the exploratory analysis revealed that just the lateral compartment held a significant difference in RTOG VAS. The addition of analysis by compartment, although leading to significance, increases the chance of Type I error. Finally, the estimated rate of grade two dermatitis was 60% in the SOC compartments. However, in this study, we found the rates were close to 41% post-treatment. Sample size estimates would indicate that we would need to increase the sample size to 77 patients to detect an improvement of 20% with the same Type I error and power. The use of BF led to non-significant reductions in patient-reported sensation scores in most categories and compartments suggesting a positive impact of BF. Likewise, Shaw et al. found a non-significant delay in pruritis when comparing BF to a corticosteroid (*p *= 0.072) and BF to no treatment (*p *= 0.079). Similar to our study, the sample size may have limited their ability to reach statistical significance [16].

Strengths of the study include an intra-individual paired design and the inclusion of patient-reported sensation scores. First, the paired design accounts for interpatient variations such as prior chemotherapy, skin-type, race, smoking status, comorbidities, and other unaccounted variables. Second, it is important to include patient-reported sensation scores because these sensations play a large role in the decision to discontinue radiotherapy [25-26]. Conventional scales such as the RTOG VAS, Common Terminology Criteria for Adverse Events, and the World Health Organization scale have low correlation with patient-reported sensations [7].

## Conclusions

In conclusion, this randomized paired comparison of BF against the SOC suggests BF may have a protective effect against radiation dermatitis on high-friction regions of the breast. BF may also reduce some common sensations associated with radiation dermatitis such as burning and pruritus. However, additional studies with slightly larger sample size and with more reliable scoring methods are needed to determine the efficacy of BF.
